# Ocular Alignment and Strabismus-Related Findings Associated with Low-Dose Atropine for Myopia Control in Children: A Structured Narrative Review

**DOI:** 10.3390/children13060818

**Published:** 2026-06-14

**Authors:** Yo Iwata, Tomoya Handa, Hitoshi Ishikawa

**Affiliations:** Department of Rehabilitation, Orthoptics and Visual Science Course, School of Allied Health Sciences, Kitasato University, 1-15-1 Kitasato, Sagamihara 252-0373, Japan

**Keywords:** low-dose atropine, myopia control, ocular alignment, binocular vision, strabismus

## Abstract

**Highlights:**

**What are the main findings?**

- Clinical and interventional studies did not show consistent worsening of ocular alignment or binocular vision with low-dose atropine.
- Case reports described temporally associated esodeviation or strabismus-related findings, particularly in children with potentially unstable binocular systems.

**What are the implications of the main findings?**

- Baseline and symptom-driven binocular vision assessment may be useful during myopia-control follow-up, especially after dose escalation.
- Case-based findings should be interpreted cautiously because incidence and causality cannot be established.

**Abstract:**

**Background/Objectives**: Low-dose atropine eye drops are widely used to slow myopia progression in children, but by reducing accommodation they may affect near ocular alignment and binocular visual function. Evidence on ocular alignment and strabismus-related findings remains insufficiently synthesized. This review examined low-dose atropine for pediatric myopia control in relation to ocular alignment and strabismus-related findings. **Methods**: PubMed/MEDLINE and Web of Science Core Collection were searched from inception to 16 April 2026. English-language studies addressing low-dose atropine, myopia control, ocular alignment, strabismus, binocular vision, accommodation, and vergence were screened. Of 247 records, 166 underwent screening after duplicate removal. Twenty-three database-derived and four manually identified full-text articles were reviewed. Eleven studies were included. **Results**: Of eleven included studies, six were clinical or interventional studies and five were case reports or case series. Case-based reports described near-predominant esodeviation, convergence excess-type deviation, elevated accommodative convergence/accommodation (AC/A) ratios, diplopia, reduced fusion, and acquired esotropia during fixed low-dose or escalating atropine use; most fixed low-dose cases improved after discontinuation or treatment modification. Clinical and interventional studies did not show consistent worsening of ocular alignment, near point of convergence (NPC), fusional vergence, or binocular vision. More consistent changes included pupil dilation, receded near point of accommodation (NPA), reduced accommodative amplitude and facility, selected fusional vergence changes, and short-term binocular or accommodative fluctuations. **Conclusions**: Low-dose atropine appears to be useful for pediatric myopia control and is generally well tolerated. However, selected cases may be temporally associated with ocular alignment abnormalities or strabismus-related findings. Careful monitoring may be warranted in children with unstable binocular systems and during dose escalation.

## 1. Introduction

The prevalence of myopia among children and adolescents has continued to rise worldwide, increasing from 24.32% in 1990 to 35.81% in 2023 and is projected to reach 39.80% by 2050, affecting more than 740 million individuals [1]. Myopia is not merely a refractive error causing blurred distance vision; it is also associated with increased risks of vision-threatening ocular diseases such as pathologic myopic maculopathy, retinal detachment, open-angle glaucoma, and cataract [2]. Pathologic myopia, in particular, is characterized by distinctive fundus lesions such as posterior staphyloma and myopic maculopathy and is recognized as a major cause of visual impairment worldwide [3]. Accordingly, suppressing myopia progression during school age and adolescence to reduce the future risk of high myopia and vision loss is a major clinical priority.

Interventions for myopia control include pharmacological treatment with low-dose atropine eye drops [4], optical approaches such as orthokeratology [5], multifocal soft contact lenses [6], and myopia-control spectacle lenses including defocus incorporated multiple segments (DIMS) lenses [7]. Among these, low-dose atropine is a pharmacological intervention with relatively robust evidence. In the LAMP study, 0.05%, 0.025%, and 0.01% atropine each significantly suppressed myopia progression and axial elongation compared with placebo at 1 year, demonstrating a concentration-dependent effect [4]. In the 5-year ATOM2 trial, 0.01% atropine showed fewer vision-related adverse effects and less rebound after cessation than higher concentrations, while maintaining a favorable long-term efficacy and safety profile [8]. Because of this balance between efficacy and tolerability, low-dose atropine has become one of the major options for pediatric myopia control, particularly in Asia.

Known adverse effects of low-dose atropine include photophobia, mydriasis, near blur related to reduced accommodation, and allergic conjunctivitis or ocular irritation [9]. In general, these adverse effects are mild, and clinical attention has focused primarily on overall efficacy and tolerability. However, because low-dose atropine can reduce accommodative responses, it may theoretically influence near ocular alignment and binocular visual function through the interaction between accommodation and convergence. Children are often exposed to substantial near visual demands from schoolwork and digital device use; therefore, even subtle functional changes may have clinical relevance to visual symptoms and treatment adherence.

Most previous studies on low-dose atropine have focused on myopia-control efficacy and general safety profiles. By contrast, evidence regarding ocular alignment, strabismus-related findings, and binocular visual changes that may be interpreted in relation to ocular alignment remains limited, heterogeneous, and not yet well synthesized, despite a growing number of case reports and small clinical studies. Therefore, organizing the available evidence on how low-dose atropine affects ocular alignment and strabismus-related findings during pediatric myopia treatment, together with binocular and accommodative changes relevant to ocular alignment, is clinically and academically important.

In this review, low-dose atropine refers to concentrations from 0.01% to 0.05%, whereas cases involving escalation beyond 0.05% are interpreted separately from fixed low-dose regimens.

Accordingly, this narrative review aimed to summarize the existing literature on the effects of low-dose atropine used for pediatric myopia control on ocular alignment and strabismus-related findings, with additional consideration of binocular visual and accommodative changes that may be interpreted in relation to ocular alignment, to clarify the current overall evidence, its clinical implications, and future research needs.

## 2. Materials and Methods

### 2.1. Review Design

This study was conducted as a structured narrative review to examine the effects of low-dose atropine eye drops used for pediatric myopia control on ocular alignment and strabismus-related findings. In this review, low-dose atropine for myopia control was operationally defined as topical atropine at concentrations from 0.01% to 0.05%, including 0.01%, 0.025%, 0.03%, and 0.05%. High-dose, or higher-concentration, atropine was defined as concentrations greater than 0.05%. Reports involving dose escalation beyond 0.05% after initiation with low-dose atropine were interpreted separately from fixed low-dose atropine regimens. Because the available literature in this field is limited and heterogeneous in study design, populations, outcomes, and follow-up duration, we performed a structured literature search aimed at a clinically relevant focused evidence synthesis rather than a systematic review predicated on quantitative pooling. A structured narrative review design was chosen because the available evidence was highly heterogeneous, including randomized trials, prospective or interventional studies, case reports, and case series with different atropine concentrations, participant characteristics, follow-up durations, and outcome measures. Case reports and case series were clinically relevant for identifying rare ocular alignment or strabismus-related findings, but they were not suitable for quantitative pooling. Therefore, this review aimed to provide a clinically focused synthesis using a structured search, study selection, and data extraction process rather than to generate a pooled estimate of effect.

### 2.2. Literature Search

PubMed/MEDLINE and Web of Science Core Collection were searched from database inception to 16 April 2026. Formal screening was restricted to English-language literature. The search strategy combined terms related to the intervention (the intervention term atropine), condition (myopia, myopia control, myopia progression, myopia management), and outcomes (ocular alignment, strabismus, esotropia, exotropia, accommodative esotropia, convergence excess, heterophoria, phoria, tropia, binocular vision, vergence, convergence, accommodation, diplopia, asthenopia, near blur). Title/Abstract fields were primarily used in PubMed, whereas Topic searching was used in Web of Science Core Collection. In addition to database searching, manual reference and citation screening was performed to identify potentially eligible articles that were not retrieved by the original database search. The complete database-specific search strategies, including exact search strings, search fields, search date, language restrictions, and the number of records retrieved, are provided in Supplementary Table S1.

### 2.3. Eligibility Criteria

Studies formally included in the focused evidence synthesis were eligible if they (1) involved children or adolescents; (2) used low-dose atropine for myopia control; and (3) reported ocular alignment, strabismus-related findings, or binocular, accommodative, or vergence measures interpretable in relation to ocular alignment. The eligible study designs included randomized controlled trials, prospective or retrospective observational studies, case series, and case reports. Case reports involving escalating atropine concentrations were considered eligible when atropine had been initiated as low-dose treatment for myopia control and ocular alignment abnormalities or strabismus-related findings were the principal outcome of the report.

Adult-only studies, studies focused mainly on high-dose or higher-concentration atropine as defined above, studies not using atropine for myopia control, studies without ocular alignment or strabismus-related outcomes, and reviews, editorials, or commentaries were excluded from formal inclusion. However, peripheral literature relevant to binocular function, accommodation, safety profiles, clinical management, or mechanistic interpretation was retained as supporting literature for the Background and Discussion sections. These supporting articles were not counted as formally included studies and were not used to derive the primary findings of the focused evidence synthesis.

### 2.4. Study Selection

**Figure 1:**
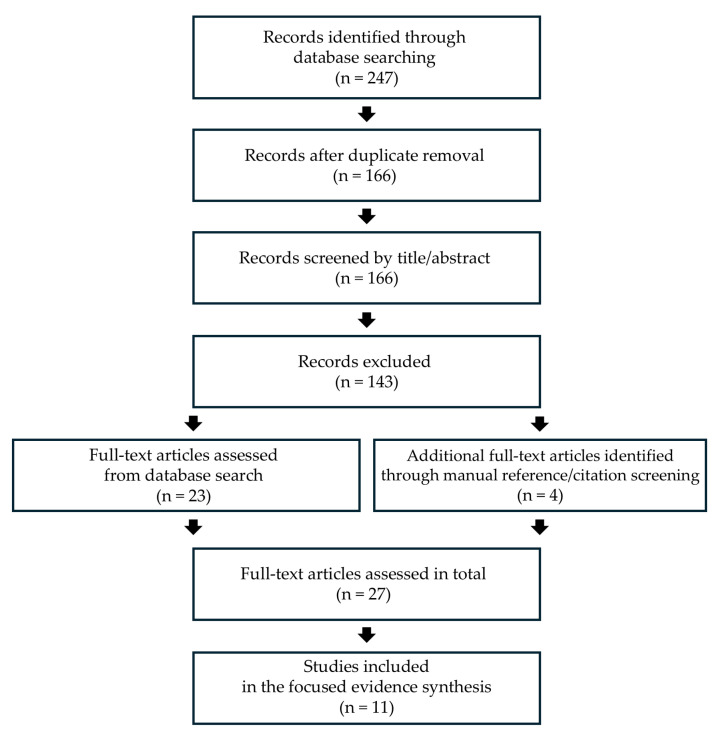
Flow diagram of literature identification, title/abstract screening, manual reference/citation screening, full-text assessment, and final inclusion of studies in the focused evidence synthesis. Of the 16 full-text articles not formally included in the focused evidence synthesis, 10 were retained as supporting literature and six were excluded after full-text assessment; the reasons for exclusion are described in Section 3.1.

After duplicate removal, title/abstract screening was performed for all identified records. Title/abstract screening and full-text assessment were conducted by three authors (Y.I., T.H. and H.I). In addition, the reference lists of key candidate and related articles were manually searched, and citation screening of relevant articles was performed to identify potentially eligible additional studies not captured by the database search. Original studies meeting the eligibility criteria were formally included in the focused evidence synthesis. The literature identification and study selection process is summarized in Figure 1.

### 2.5. Data Extraction and Synthesis

Data extracted from the included studies comprised study design, participant characteristics, atropine concentration, treatment purpose, follow-up duration, ocular alignment outcomes, strabismus-related findings, binocular and accommodative measures interpretable in relation to ocular alignment, main findings, and major methodological limitations.

Because the study design, outcome measures, follow-up duration, and study population were heterogeneous, no quantitative synthesis was performed. Instead, the evidence was narratively synthesized with emphasis on (1) evidence directly addressing ocular alignment and strabismus-related findings, (2) binocular and accommodative changes interpretable in relation to ocular alignment, and (3) clinical implications and study limitations. The extracted study characteristics, including study design, participant age, atropine regimen, follow-up or assessment window, baseline assessment, evaluated parameters, and main findings, were summarized descriptively in Table 1.

### 2.6. Methodological Appraisal

Because the included studies were heterogeneous and included randomized trials, prospective or interventional studies, case reports, and case series, we did not apply a single formal risk-of-bias instrument across all studies, such as Cochrane RoB 2, the Newcastle-Ottawa Scale, or JBI critical appraisal tools. Instead, we conducted a structured qualitative methodological appraisal based on study design, sample size, presence of a control group, prospective or retrospective data collection, baseline assessment of ocular alignment or binocular vision, follow-up duration, and whether ocular alignment or binocular vision was a primary or secondary outcome. Case reports and case series were considered hypothesis-generating evidence and were interpreted separately from randomized and prospective clinical studies. The results of this qualitative methodological appraisal are summarized in the Results section.

## 3. Results

### 3.1. Literature Search Results

The database search identified 109 records from PubMed/MEDLINE and 138 from the Web of Science Core Collection, for a total of 247 records. After import into the reference management software and removal of duplicates, 166 records underwent title/abstract screening. Of these, 143 were excluded at the title/abstract stage and 23 proceeded to a full-text review. An additional four articles were identified through manual reference and citation screening. Accordingly, 27 full-text articles were assessed, of which eleven original studies were formally included in the focused evidence synthesis. All four studies identified through manual reference/citation screening were ultimately included (Figure 1). Of the 16 full-text articles that were not formally included in the focused evidence synthesis, 10 were retained as supporting literature for mechanistic interpretation, broader safety context, clinical management discussion, or contextual interpretation. These supporting articles were not used to derive the primary findings of the focused evidence synthesis. The remaining six articles were excluded after full-text assessment because they did not meet the eligibility criteria, mainly due to insufficient ocular alignment, strabismus-related, binocular vision, accommodative, or vergence outcomes interpretable in relation to ocular alignment, lack of a suitable pediatric low-dose atropine myopia-control context, or a combined optical/pharmacologic intervention design that did not allow the atropine-specific ocular alignment or strabismus-related effect to be evaluated.

### 3.2. Characteristics of the Included Studies

Of the eleven included studies, six were clinical or interventional studies and five were case reports or case series. The clinical or interventional studies comprised a placebo-controlled randomized clinical trial in children with basic-type intermittent exotropia (IXT), a prospective randomized controlled trial that included a combination of 0.01% atropine and orthokeratology (OK), a prospective case–control study of 0.01% atropine, a randomized double-masked study examining short-term changes after a single instillation of 0.01–0.05% atropine, a prospective study following three 0.05% atropine regimens for 12 months, and a randomized interventional study evaluating accommodation and vergence after 14 nights of 0.025% or 0.05% atropine. The concentrations evaluated were mainly 0.01%, 0.025%, and 0.05%, although one case report involved escalating atropine concentrations initiated as low-dose treatment.

Outcomes included distance and near ocular alignment, near and distance phoria, magnitude of exodeviation, accommodative convergence/accommodation (AC/A) ratio, near point of convergence (NPC), fusional vergence, accommodative amplitude, accommodative facility, accommodative lag, and pupil size. The follow-up or assessment windows ranged from 24 h after a single instillation to 12 months in the clinical and interventional studies, whereas case reports described ocular alignment abnormalities occurring from approximately 1 month to longer-term treatment periods, including one case involving escalating atropine concentrations [10,11,12,13,14,15,16,17,18,19,20].

Among the six clinical or interventional studies, participant ages ranged from 6 to 17 years. Baseline ocular alignment, binocular vision, accommodative function, or vergence-related parameters were assessed in all six studies, although the specific test battery differed across studies. Assessment timing also varied substantially. The AMIXT randomized clinical trial assessed ocular alignment, exotropia control, stereoacuity, NPC, AC/A ratio, and fusional vergence repeatedly during 12 months. Jiang et al. assessed accommodation and vergence function at baseline and 3 months. Neena et al. evaluated ocular alignment, near point of accommodation, and NPC at treatment initiation, 1 month, and then every 6 months, with a minimum follow-up of 6 months. Breliant et al. assessed binocular vision and accommodation at baseline and 30 min, 60 min, and 24 h after a single instillation. Pan et al. assessed accommodation and binocular vision at baseline, week 2, month 6, and month 12. Santos-Neto et al. evaluated accommodation and vergence parameters at baseline and on the morning of day 15 after 14 nights of atropine use. These study-specific details, including participant age, follow-up duration or assessment window, baseline assessment, evaluated parameters, and main findings, are summarized in Table 1.

To avoid ambiguity, only the eleven studies listed in Table 1 were treated as formally included studies in the focused evidence synthesis. Other articles cited in the Background or Discussion were used as supporting literature only, to provide mechanistic context, broader safety information, or clinical management considerations. These supporting articles did not contribute to the primary synthesis of included-study findings. This distinction is summarized in Table 2.

Because the included studies differed substantially in design, sample size, control conditions, follow-up duration, and whether ocular alignment or binocular vision was a primary outcome, their interpretive weight was not considered equivalent. A qualitative methodological appraisal of the formally included studies is summarized in Table 3.

### 3.3. Studies Directly Reporting Ocular Alignment and Strabismus-Related Findings

#### 3.3.1. Esodeviation

In case reports and case series, near-predominant esodeviation or convergence excess-type deviation was reported during low-dose atropine use. In the case reported by Iwata et al., an 8-year-old boy developed intermittent esotropia of 12 prism diopters (PD) at near and esophoria of 2 PD at distance 1 month after the initiation of 0.025% atropine. The gradient AC/A ratio increased to 8 PD/D at distance and 6.7 PD/D at near. With +3.00 D near add, near alignment shifted to 8 PD exophoria, and by 28 d after discontinuation, near alignment had improved to 2 PD exophoria, whereas the AC/A ratio decreased to 4 PD/D at distance and 2 PD/D at near. Near visual acuity, near alignment, and the AC/A ratio improved over time after discontinuation [16].

Kothari et al. reported convergence excess consecutive esotropia during 0.01% atropine administration in three children after surgery for intermittent exotropia. The mean age was 5.3 ± 1.2 years, mean near esodeviation was +28.3 PD, and mean distance esodeviation was +10.6 PD. All three patients showed a high AC/A ratio, and fusion recovered within three weeks after discontinuation. Improvement in positive relative accommodation and accommodative facility was also documented in at least two cases [17].

In the case reported by Jahan et al., a 10-year-old boy with progressive myopia was switched from 1% atropine to 0.01% atropine and developed poorly controlled near esophoria of +16 PD after six months, together with an additional −0.50 D of myopic progression in both eyes. The original report showed renewed myopia progression during 0.01% atropine use, followed by stabilization after restarting 1% atropine. Three weeks after discontinuation of 0.01% atropine, accommodation, convergence, and esodeviation had normalized, and nine months after resuming 1% atropine, both near and distance alignments were stable, as exophoria and sensory fusion had been re-established [18].

Belbase et al. described an 8-year-old boy who developed intermittent esotropia, measuring 18 PD at near and 6 PD at a distance, three months after the initiation of 0.01% atropine. The AC/A ratio was high (7:1), and uncrossed diplopia at both distance and near, together with loss of stereoacuity, was documented using the Worth four-dot test. The original report showed that the initial near exophoria of 2 PD and orthophoria at distance changed to 18 PD esotropia at near and 6 PD esophoria at distance during 0.01% atropine use, followed by improvement to 2 PD esophoria at near and 4 PD exophoria at distance after switching to multifocal soft contact lenses [19].

Aman and Guyton reported a 15-year-old boy who developed acquired esotropia during long-term atropine treatment for rapidly progressive myopia. The authors interpreted the esotropia as a rare complication of escalating atropine concentrations, presumably related to atropine-induced accommodative duress and excessive convergence. Because this report involved escalating atropine concentrations rather than fixed low-dose monotherapy, it should be interpreted separately from cases occurring during stable 0.01–0.05% treatment [20].

Overall, the case-based literature has repeatedly documented near-predominant esodeviation, convergence excess-type deviation, elevated AC/A ratio, diplopia, reduced fusion, and acquired esotropia during fixed low-dose or escalating atropine use. In most reports involving fixed low-dose atropine, ocular alignment and binocular visual function improved after discontinuation of low-dose atropine or after a change in treatment, whereas the case involving long-term escalating concentrations should be interpreted separately [16,17,18,19,20].

#### 3.3.2. Intermittent Exotropia

In contrast, the AMIXT randomized clinical trial in children with IXT showed that 0.01% atropine significantly suppressed myopia progression and axial elongation without adverse effects on exotropia conditions or binocular vision. A total of 300 children were enrolled. At 1 year, the change in cycloplegic spherical equivalent was −0.51 D in the atropine group and −0.75 D in the placebo group, a between-group difference of 0.24 D. Axial elongation was 0.31 mm in the atropine group and 0.42 mm in the placebo group, a difference of −0.11 mm. Regarding ocular alignment-related findings, the magnitude of near exodeviation decreased in the atropine group but increased in the placebo group (−1.25 PD vs. 0.74 PD; difference, −1.99 PD; P = 0.03). The frequencies of photophobia and blurred near vision were not substantially different from those in the placebo group. Thus, at least in children with stable basic-type IXT, 0.01% atropine did not appear to worsen exotropia control [10]. Because AMIXT was the largest and most rigorously designed study included in this review, with a placebo-controlled randomized design, 300 enrolled children, 12-month follow-up, and direct assessment of exotropia control and binocular vision, its findings should be given greater interpretive weight than isolated case reports. These data suggest that, in children with stable basic-type intermittent exotropia, 0.01% atropine did not produce adverse binocular vision effects or deterioration of exotropia condition at the group level.

### 3.4. Studies Evaluating Binocular and Accommodative Parameters Relevant to Ocular Alignment

#### 3.4.1. Binocular Vision, Accommodation, and Fusional Vergence

In the prospective case–control study by Neena et al., 36 Indian children (71 eyes) using 0.01% atropine were compared with 19 age-matched controls (37 eyes). At six months, the 0.01% atropine group showed significantly less myopia progression and axial elongation than the control group, whereas no significant changes were observed in ocular alignment or NPC. The observed changes were an average increase in pupil diameter of 0.83 mm and a 1.14 cm recession of the near point of accommodation (NPA), both of which were considered clinically mild [12].

In a prospective randomized controlled trial by Jiang et al., children were assigned to receive 0.01% atropine alone, OK alone, 0.01% atropine plus OK, or control; 62 children completed the 3-month evaluation. At baseline, there were no between-group differences in the accommodative or vergence indices. At three months, a significant decrease in accommodative lag was observed only in the OK group. The binocular accommodative facility and positive relative accommodation increased in the combination and OK groups, but no significant changes were observed in the 0.01% atropine alone group. In addition, no between-group differences were found for changes in fusional vergence or the AC/A ratio, and no clear deterioration in vergence parameters attributable to 0.01% atropine was demonstrated [11].

In a randomized double-blind study by Breliant et al., 46 children received a single instillation of placebo or 0.01%, 0.03%, or 0.05% atropine and underwent binocular vision and accommodation assessments at 30 min, 60 min, and 24 h. No significant changes compared to placebo were found in dissociated phoria, NPC, fusional vergence, accommodative lag, accommodative amplitude, or distance/near visual acuity at any atropine concentration. By contrast, pupil size significantly increased in the 0.03% and 0.05% groups, whereas only a minimal change at the 60 min scotopic time point was observed in the 0.01% group [13].

#### 3.4.2. Studies Showing Transient Binocular/Accommodative Changes Without Reported Strabismus

Pan et al. reported a secondary analysis of a 12-month prospective study comparing once-daily, twice-weekly, and once-weekly regimens of 0.05% atropine. A total of 205 children were analyzed, and accommodation and binocular vision were assessed at week 2, month 6, and month 12. Short-term changes in accommodation and binocular vision were observed in all groups; however, most of these changes had returned to baseline by month 12. For example, in the once-daily group, the AC/A ratio increased from 5.13 PD/D at baseline to 5.82 PD/D at week 2 and then decreased to 5.22 PD/D at month 12. No cases of strabismus were diagnosed or reported during the study period [14].

Santos-Neto et al. evaluated accommodation and vergence parameters in 34 myopic schoolchildren aged 7–17 years after 14 nights of 0.025% or 0.05% atropine. Significant accommodative changes were observed in both groups, including recession of the NPA, reduction in accommodative amplitude, reduction in accommodative facility, reduction in PRA in absolute terms, and increased pupillary diameter. The authors also reported significant vergence changes limited mainly to near PFV with 0.025% atropine and far PFV with both 0.025% and 0.05% atropine. Under the conditions of that study, 0.025% and 0.05% atropine were associated with measurable accommodative disturbance and visual discomfort, although manifest strabismus was not reported [15].

## 4. Discussion

### 4.1. Discrepancy Between Case-Based Reports and Comparative Studies

In this narrative review, we synthesized eleven original studies on the effects of low-dose atropine, used for pediatric myopia control, on ocular alignment and strabismus-related findings, together with binocular and accommodative changes interpretable in relation to ocular alignment. Current studies suggest that low-dose atropine may, in selected cases, be temporally associated with ocular alignment abnormalities or strabismus-related findings, whereas clinical and interventional studies have not shown consistent worsening of ocular alignment, leaving the evidence limited and heterogeneous. In other words, while case reports and case series described near-predominant esodeviation, convergence excess-type deviation, elevated AC/A ratio, diplopia, reduced fusion, and acquired esotropia, clinical and interventional studies generally did not demonstrate clear deterioration in ocular alignment, fusional vergence, NPC, or binocular vision overall [10,11,12,13,14,15,16,17,18,19,20]. Among the included studies, the AMIXT placebo-controlled randomized clinical trial provides the strongest group-level evidence and did not demonstrate adverse effects of 0.01% atropine on exotropia condition or binocular vision in children with stable basic-type intermittent exotropia.

Importantly, as summarized in Table 3, the evidentiary weight of the included studies was not equivalent. The randomized and prospective clinical studies provide more reliable information on group-level effects, whereas case reports and case series are useful for detecting rare or clinically unusual events but are inherently subject to publication and selection bias. Therefore, the case-based evidence should be interpreted as hypothesis-generating and cannot be used to estimate the incidence of ocular alignment abnormalities or to establish a causal relationship with low-dose atropine.

Several factors may explain this discrepancy between case-based reports showing abnormalities and comparative studies showing no consistent worsening. First, the patients highlighted in the case reports were likely to have a vulnerable binocular system at baseline. The three cases reported by Kothari et al. were all postoperative intermittent exotropia cases, and the authors themselves suggested tenuous fusion or a monofixation-like state [17]. In the case reported by Jahan et al., flick esophoria at near was already present before the introduction of 0.01% atropine, whereas in the case reported by Belbase et al., initial near exophoria was followed by esotropia accompanied by a high AC/A ratio and binocular diplopia after treatment [18,19]. In the case reported by Iwata et al., baseline near exophoria of 2 PD became symptomatic near-predominant esotropia after initiation of 0.025% atropine, with a concomitant increase in the AC/A ratio [16]. Thus, these case reports do not demonstrate that low-dose atropine caused new ocular alignment changes. Rather, they suggest that ocular alignment abnormalities were observed in children who may already have had vulnerable or unstable binocular systems, and that low-dose atropine use was temporally associated with the clinical presentation in these selected cases. Aman and Guyton further extended this case-based pattern by describing acquired esotropia in a 15-year-old boy during long-term atropine treatment with escalating concentrations. This case is mechanistically relevant because the authors attributed the deviation to accommodative duress and excessive convergence, but it should be interpreted cautiously because the exposure was not fixed low-dose monotherapy [20].

### 4.2. Exclusion of High-Risk Binocular Populations from Comparative Studies

Second, many comparative studies have deliberately excluded high-risk cases. In the study by Breliant et al., children with strabismus, amblyopia, previous strabismus surgery, or prior vision therapy were excluded, meaning that participants with overt binocular instability were excluded [13]. Similarly, Pan et al. excluded children with abnormal binocular function or strabismus [14]. In a case–control study by Neena et al., ocular alignment and NPC were assessed; however, the cohort represented more typical Indian children with progressive myopia than a specific high-risk binocular population [12]. Accordingly, these studies are informative about the average effects of low-dose atropine in general pediatric myopia populations. However, they did not adequately evaluate the risk in patients with latent strabismus, high AC/A ratios, prior strabismus surgery, or fragile fusional reserves. Moreover, although Jiang et al. measured binocular function in some detail, their primary aim was to compare orthokeratology-based regimens, and the follow-up was limited to 3 months [11]. Therefore, the absence of consistent worsening in comparative studies should not be interpreted as evidence that risk is absent.

### 4.3. Heterogeneity in Atropine Concentration, Outcome Measures, and Assessment Timing

Third, differences in outcome measures and assessment timing likely contributed to between-study inconsistencies. Although baseline ocular alignment, binocular vision, accommodative function, or vergence-related parameters were assessed in the clinical and interventional studies, the specific test batteries and follow-up intervals differed substantially among studies. Therefore, comparisons across prospective studies should be interpreted cautiously, because the ability to detect ocular alignment or binocular vision changes may depend on both the parameters assessed and the timing of follow-up. Breliant et al. examined short-term changes at 30 min, 60 min, and 24 h after a single instillation of 0.01%, 0.03%, or 0.05% atropine, and found no significant changes in phoria, NPC, fusional vergence, accommodative lag, or accommodative amplitude [13]. In contrast, in the 0.05% study by Pan et al., short-term changes in accommodation and binocular vision, including the AC/A ratio, were observed at week 2, although most approached baseline by month 12 [14]. In addition, in the cases reported by Iwata et al. and Belbase et al., symptomatic ocular alignment abnormalities emerged relatively early, within 1–3 months after the initiation of low-dose atropine [16,19]. Thus, the effects of low-dose atropine on the binocular system may be transient and subclinical in some individuals but overtly symptomatic in others, making it difficult to capture the overall picture at a single time point.

### 4.4. Dose-Related Accommodative and Vergence Responses

One possible mechanistic interpretation is that partial cycloplegia may alter accommodative demand during near tasks, potentially affecting accommodative convergence in susceptible children. In a case reported by Iwata et al., a transient increase in the AC/A ratio was documented in parallel with near-predominant esotropia, and both improved after discontinuation [16]. Similarly, Kothari et al. documented high AC/A ratios and proposed that an excessive innervational drive to compensate for hypoaccommodation may have produced manifest esotropia [17]. Belbase et al. reported a high AC/A ratio of 7:1 accompanied by binocular diplopia and loss of stereoacuity [19]. In contrast, group-level deterioration in the AC/A ratio or vergence parameters was not demonstrated in the studies by Neena et al. or Jiang et al. [11,12]. In Pan et al., short-term AC/A elevation was observed but tended to return toward baseline over time [14]. These findings suggest that the influence of low-dose atropine on the accommodation-convergence system is not uniform across all patients, but may depend on each child’s binocular status and accommodative reserve. This interpretation is further supported by Santos-Neto et al., who showed that 0.025% and 0.05% atropine could reduce accommodative amplitude, recede the NPA, reduce accommodative facility, and alter selected PFV parameters after 14 nights of use [15].

This interpretation is further informed by supporting literature outside the eleven formally included studies. Woodman-Pieterse et al. reported that in young adults using 0.05% atropine for 9 consecutive nights, accommodative lag increased; accommodative amplitude, positive relative accommodation, and binocular accommodative facility decreased; near heterophoria shifted significantly in the esophoric direction; and negative fusional vergence break and recovery declined by day 10 [21]. Although no clear change in distance heterophoria or AC/A ratio was found, the overall pattern—greater convergence demand near with reduced divergence reserve—is consistent with the near-predominant esodeviation observed in previous case reports [21]. Thus, even when manifest strabismus does not develop, these findings are compatible with short-term shifts in near binocular or vergence demand during atropine exposure. However, they should not be interpreted as evidence that low-dose atropine independently induces clinically significant esodeviation.

Hughes et al. further showed that 1 week of 0.025% atropine reduced accommodation-induced changes in anterior chamber depth and lens thickness and reduced the accommodative response itself [22]. Mitsukawa et al. similarly used swept-source anterior segment optical coherence tomography after a single instillation of 0.01% atropine and detected changes in the anterior chamber depth and posterior lens curvature under a 5 D accommodative stimulus, suggesting a subtle reduction in accommodation [23]. Although these studies focused on morphological and functional accommodative changes rather than ocular alignment, they indicated that it is overly simplistic to assume that low-dose atropine produces negligible cycloplegia. Even low-dose atropine may slightly reduce the accommodative response required for near work in some individuals, potentially producing secondary effects on the vergence system [21,22,23].

### 4.5. Responses in Intermittent Exotropia and Esodeviation-Prone Patients

Simultaneously, it is important to note that 0.01% atropine does not appear to be uniformly disadvantageous for all strabismus-related conditions. In a large randomized clinical trial, AMIXT, conducted in children with basic-type IXT, 0.01% atropine not only slowed myopia progression, but also reduced the magnitude of near exodeviation relative to placebo [10]. No adverse effect on exotropia conditions or binocular vision was observed [10]. In addition, supporting literature outside the formal focused evidence synthesis included a randomized study by Dehghanian Nasrabadi et al., which reported that 0.05% low-dose atropine improved deviation control in intermittent exotropia and showed efficacy comparable to overminus lenses [24]. These findings are crucial to avoid the oversimplification that low-dose atropine is uniformly dangerous for children with strabismus. In stable IXT, 0.01% or 0.05% low-dose atropine may not worsen ocular alignment and may even be beneficial for exodeviation control. However, AMIXT was limited to basic-type IXT [10], and the study by Dehghanian Nasrabadi et al. evaluated deviation control over only three months [24]. Therefore, these findings cannot be directly generalized to postoperative exotropia, esophoria, high AC/A ratios, or symptomatic near-work-related binocular stress.

### 4.6. Clinical Monitoring, Low-Dose Atropine, and Binocular-Vision Exclusion Criteria

Clinically, this means that low-dose atropine should not be prescribed as though all children respond similarly; rather, it is important to identify patients who warrant closer binocular monitoring. Based on the current evidence, regular binocular vision and strabismus-related assessment should be considered as part of myopia-control follow-up, particularly at treatment initiation, after dose escalation, and when symptoms such as near blur, diplopia, asthenopia, or reduced fusion develop. However, the current literature does not support a specific high-risk age group. Instead, higher-risk profiles appear to be defined more by baseline binocular status and ocular history than by age alone. Particular caution seems advisable in (1) patients with a history of strabismus surgery, (2) children with esophoria or latent esodeviation at near, (3) those with a high AC/A ratio at baseline or an increasing AC/A ratio shortly after treatment initiation, and (4) those with unstable fusional ability or those who develop near blur, diplopia, asthenopia, or reduced fusion soon after starting low-dose atropine [16,17,18,19,20]. In contrast, stable basic-type IXT does not necessarily appear to be a contraindication for 0.01% atropine [10,24]. Accordingly, before initiating low-dose atropine, it may be useful to evaluate distance and near deviation, phoria, NPC, stereoacuity, and when appropriate, the AC/A ratio. If symptoms arise after treatment initiation, a similar binocular assessment should be promptly repeated. If symptomatic near blur, diplopia, asthenopia, reduced fusion, or progression of near esodeviation develops, dose reduction, temporary discontinuation, near addition lenses, or a switch to optical myopia control strategies should be considered [16,17,18,19,25].

### 4.7. Discontinuation, near Addition, and Treatment Modification

In this regard, supporting literature on near visual support provides practical clinical guidance. Sun et al. reported that adding progressive addition lenses (PALs) to children receiving topical atropine improved the distance and near visual acuity, accommodative lag, binocular visual function, and daily visual complaints [25]. This study also suggested that heterophoria measured at the Harmon distance shifted in a more exo direction through near addition, emphasizing the importance of routinely following stereopsis and NPC [25]. Similarly, in a case reported by Belbase et al., binocular fusion recovered after discontinuing 0.01% atropine and switching to multifocal soft contact lenses [19]. These findings suggest that when near symptoms or binocular stress emerge during low-dose atropine treatment, near addition or switching to optical myopia control strategies may be realistic management options.

### 4.8. Atropine Concentration, Iris Pigmentation, and Individual Variability

Dose-dependence and possible differences across populations or iris pigmentation warrant attention. Joachimsen et al. reported that in German schoolchildren, 0.05% atropine produced greater anisocoria and reduced accommodative amplitude than 0.01% atropine, and 63% of the participants reported visual impairment or reading difficulties [26]. In contrast, in a Caucasian population using 0.01% atropine, Loughman et al. found changes in pupil size and responsiveness, but minimal effects on distance or near visual acuity, reading speed, or quality of life, and all participants considered the drops tolerable [27]. In the Indian pediatric randomized trial by Janti et al., 0.05% showed greater efficacy than 0.01%, while both groups exhibited increased NPA and pupil size; these changes were greater in the 0.05% group, although adverse effects were mild and did not substantially compromise compliance [28]. The study by Santos-Neto et al. reinforces this dose-related concern because both 0.025% and 0.05% atropine produced significant accommodative disturbances after 14 nights, with reductions in accommodative amplitude and accommodative facility and increases in pupil diameter [15]. Together, these findings suggest that the severity of binocular side effects and near vision symptoms may be modified by concentration, study population, iris characteristics, or accommodative reserve. Therefore, children who tolerate 0.01% without difficulty cannot be assumed to respond similarly to 0.025% or 0.05%, and attention to binocular symptoms is warranted when concentration is increased.

In the broader atropine literature, worsening of binocular function has not been uniformly reported. In a prospective study of Nepalese children, Adhikari et al. found no meaningful changes in near vision or near the point of accommodation and reported very few ocular or systemic side effects [29]. Kuo et al., in Taiwanese schoolchildren, documented increases in pupil size, reductions in distance and near visual acuity, reduced accommodation, reduced convergence ability, reduced stereopsis, and more visual symptoms in the atropine group; however, that study included relatively higher concentrations such as 0.125–0.5% [30], and therefore does not directly represent current low-dose atropine practice (0.01–0.05%). Nonetheless, this suggests a general dose-related trend in which binocular function and daily visual performance are more likely to be affected as atropine concentration increases.

### 4.9. Strengths and Limitations

A strength of this review is that it used a structured search strategy, clearly distinguished formally included studies from supporting literature, and appraised the interpretive weight of heterogeneous evidence. In addition, the review integrates randomized and prospective clinical studies with case-based reports while explicitly avoiding causal overinterpretation. This review has several limitations. First, only eleven studies were included, five of which were case reports or case series, precluding a robust estimation of incidence or causal inference. These case-based reports are also susceptible to publication and selection bias, because unusual or symptomatic ocular alignment events are more likely to be reported than uneventful atropine use. In addition, several affected children had pre-existing or potentially vulnerable binocular conditions, such as prior strabismus surgery, latent esophoria, high AC/A ratio, or unstable fusional ability; therefore, the observed events cannot be attributed to atropine exposure alone. Second, in many comparative studies, ocular alignment or binocular function was not the primary outcome, and the measurement methods and follow-up durations varied considerably [11,12,13,14,15]. In addition, attrition or dropout in some included studies may have affected interpretation because reasons for discontinuation were not consistently reported or were not always categorized according to visual discomfort, accommodative symptoms, diplopia, asthenopia, near blur, or binocular vision problems. Therefore, treatment-related near-vision or binocular symptoms may have been underestimated, particularly in studies with substantial attrition. Third, the study populations were diverse, including general pediatric myopia populations, orthokeratology combination regimens, children with IXT, and postoperative exotropia cases, limiting direct between-study comparison [10,11,12,13,14,15,16,17,18,19,20]. In addition, one case report involved escalating atropine concentrations, rather than fixed 0.01–0.05% monotherapy; therefore, it should not be interpreted as direct evidence of risk during standard fixed low-dose regimens [20]. Fourth, this was itself an English-language narrative review and therefore remains subject to selection bias in the search strategy, eligibility decisions, and interpretation. In addition, some supporting discussion literature includes mechanistic studies in adults and observational studies involving different populations or higher atropine concentrations [21,22,23,27,30], and extrapolation of these findings to school-aged children should be performed cautiously. Accordingly, this review aimed to organize the current evidence and highlight its clinical implications rather than to determine the incidence or establish a definitive risk stratification.

### 4.10. Future Research

Future research should prospectively and systematically evaluate the ocular alignment and strabismus-related findings using standardized methods. Specifically, longitudinal measurements of distance and near deviation, phoria, NPC, fusional vergence, stereoacuity, AC/A ratio, and near visual symptoms should be performed across commonly used concentrations such as 0.01%, 0.025%, and 0.05%. It is also important to stratify potentially high-risk groups, including those with postoperative exotropia, latent esophoria, high AC/A cases, and children with a high near-work demand. In addition, future studies should clarify how low-dose atropine-related binocular side effects affect treatment adherence and patient-reported outcomes and to what extent near additions or multifocal optical strategies can mitigate such problems [19,25].

Overall, the included studies indicated that low-dose atropine eye drops are generally useful for pediatric myopia control and that clinical and interventional studies have not demonstrated consistent worsening of ocular alignment. Simultaneously, selected cases may develop near-predominant esodeviation, convergence excess-type deviation, or acquired esotropia. When the broader supporting literature is also considered, low-dose atropine may be associated with changes in accommodative response and near-vergence demand, particularly at 0.025% and 0.05%, but the clinical phenotype likely varies according to concentration, study population, baseline binocular status, prior ocular history, and dose escalation. Thus, low-dose atropine should not be viewed as uniformly “safe” or uniformly “dangerous”; rather, it is best understood as a generally well-tolerated treatment that nevertheless requires careful initiation, dose escalation, and follow-up in a subset of children with potentially unstable binocular systems.

## 5. Conclusions

This narrative review suggests that low-dose atropine eye drops are useful for pediatric myopia control and are generally well tolerated in many children, although selected cases may be temporally associated with ocular alignment abnormalities or strabismus-related findings. Clinical and interventional studies, particularly the AMIXT randomized clinical trial, have not demonstrated consistent worsening of ocular alignment or binocular vision; however, case reports have described near-predominant esodeviation, convergence excess-type deviation, elevated AC/A ratios, reduced fusion, and acquired esotropia. Therefore, case-based findings should be interpreted as clinically important but hypothesis-generating observations rather than evidence of a group-level adverse binocular vision effect. In addition, short-term interventional data indicate that 0.025% and 0.05% atropine can measurably affect accommodation and selected vergence parameters. Careful initiation, dose escalation, and follow-up are particularly important in children with unstable binocular systems. Prospective studies using standardized assessments of ocular alignment, binocular function, accommodation, vergence, and visual symptoms are needed. Current evidence is insufficient to determine incidence or establish causality, and case-based findings should be interpreted in the context of baseline binocular vulnerability and the stronger evidence from prospective clinical studies.

## Figures and Tables

**Table 1:** Study characteristics, follow-up duration or assessment window, baseline assessment, and main ocular alignment/binocular vision findings of the included studies.

Study	Design/ Participants	Age	Atropine Regimen	Follow-Up or Assessment Window	Baseline Assessment/Parameters	Main Findings
**Clinical and interventional studies**						
Wang et al., 2024 (AMIXT) [10]	Placebo-controlled RCT; 300 children with myopia and basic-type IXT	6–12 years	0.01% once nightly for 12 months	12 months; repeated assessments during follow-up	Baseline and repeated assessments over 12 months: alignment, exotropia control, stereoacuity, NPC, AC/A ratio, fusional vergence	Near exodeviation decreased in the atropine group; no adverse effect on exotropia condition or binocular vision
Jiang et al., 2023 [11]	Prospective RCT; 62 children completed evaluation	8–12 years	0.01% alone, 0.01% + OK, OK alone, or control for 3 months	3 months	Baseline and 3 months: accommodation and vergence function	No obvious deterioration in vergence parameters or AC/A ratio in the atropine-alone group
Neena et al., 2022 [12]	Prospective case–control study; 36 atropine-treated children and 19 controls	6–16 years	0.01% once nightly; minimum follow-up 6 months	Minimum 6 months; treatment initiation, 1 month, then every 6 months	Treatment initiation, 1 month, then every 6 months: ocular alignment, NPA, NPC	No significant changes in ocular alignment or NPC; mild pupil dilation and NPA recession
Breliant et al., 2023 [13]	Randomized double-masked study; 46 children	6–17 years	Single instillation of placebo, 0.01%, 0.03%, or 0.05%	Single instillation; 30 min, 60 min, and 24 h	Baseline, 30 min, 60 min, and 24 h: phoria, NPC, fusional vergence, accommodation	No significant changes in phoria, NPC, fusional vergence, or accommodation; pupil size increased mainly with 0.03% and 0.05%
Pan et al., 2025 [14]	Secondary analysis of a prospective study; 205 children	6–14 years	0.05% once daily, twice weekly, or once weekly for 12 months	12 months; baseline, week 2, month 6, and month 12	Baseline, week 2, month 6, and month 12: accommodation and binocular vision	Short-term accommodation/binocular vision changes were observed, but most returned close to baseline by 12 months; no strabismus
Santos-Neto et al., 2025 [15]	Randomized interventional study; 34 myopic schoolchildren	7–17 years	0.025% or 0.05% once nightly for 14 nights	14 nights; assessed on the morning of day 15	Baseline and morning of day 15: accommodation and vergence parameters	Accommodative function worsened and limited PFV changes were observed; no manifest strabismus
**Case reports and case series**						
Iwata et al., 2026 [16]	Case report; 1 child	8 years	0.025% once nightly	Baseline, 1 month after initiation, and after discontinuation	Baseline, 1 month after initiation, and after discontinuation: ocular alignment and AC/A ratio	Near intermittent esotropia and elevated AC/A ratio developed after initiation and improved after discontinuation
Kothari et al., 2020 [17]	Case series; 3 children after surgery for IXT	4–6 years	0.01% for 4–16 months	4–16 months of atropine use; recovery after discontinuation described	Pre-event clinical findings and post-discontinuation course were described	Convergence excess consecutive esotropia with high AC/A ratio occurred; fusion recovered after discontinuation
Jahan et al., 2021 [18]	Case report; 1 child	10 years	0.01% after switch from 1% atropine	Clinical course before, during, and after 0.01% atropine described	Clinical course before, during, and after 0.01% atropine was described	Near esophoria and rebound myopia occurred during 0.01% use and normalized after discontinuation
Belbase et al., 2025 [19]	Case report; 1 child	8 years	0.01% for 3 months	Baseline, 3 months after initiation, and after treatment change	Baseline, 3 months after initiation, and after treatment change were described	Intermittent esotropia with high AC/A ratio, diplopia, and reduced stereopsis developed; fusion improved after discontinuation and treatment change
Aman and Guyton 2025 [20]	Case report; 1 adolescent	15 years	Escalating concentrations after initiation with low-dose atropine	Long-term clinical course during dose escalation	Long-term clinical course described; not a fixed low-dose regimen	Acquired esotropia developed during long-term dose escalation; interpreted separately from fixed low-dose regimens

Abbreviations: AC/A, accommodative convergence/accommodation; IXT, intermittent exotropia; NPC, near point of convergence; NPA, near point of accommodation; OK, orthokeratology; PFV, positive fusional vergence; RCT, randomized controlled trial.

**Table 2:** Distinction between formally included studies and supporting literature.

Evidence Category	References	Role in this Review
Formally included studies	[10,11,12,13,14,15,16,17,18,19,20]	Contributed to the focused evidence synthesis, Results, Table 1, and main conclusions regarding ocular alignment, strabismus-related findings, binocular vision, accommodation, or vergence outcomes in children using low-dose atropine for myopia control.
Supporting mechanistic literature	[21,22,23]	Used only to inform mechanistic interpretation of accommodative or vergence-related changes; not counted as formally included evidence.
Supporting clinical or contextual literature	[24,25,26,27,28,29,30]	Used only to provide broader clinical context, management considerations, dose-related safety information, or comparison with related atropine literature; not counted as formally included evidence.

**Table 3:** Qualitative methodological appraisal of the formally included studies.

Study	Study Design/Sample Size	Main Methodological Strengths	Main Limitations/Interpretive Weight
Wang et al., 2024 (AMIXT) [10]	Placebo-controlled randomized clinical trial; *n* = 300	Randomized design, placebo control, large sample size, 12-month follow-up, and direct assessment of exotropia condition and binocular vision	Strongest included evidence for group-level safety in children with stable basic-type intermittent exotropia; generalizability to esophoria, high AC/A ratio, postoperative strabismus, or unstable binocular systems is limited
Jiang et al., 2023 [11]	Prospective randomized controlled trial; *n* = 62 completed evaluation	Prospective design, comparator groups, and assessment of accommodation and vergence function at baseline and follow-up	Short follow-up duration; atropine-alone group was not the sole focus; limited power for rare ocular alignment events
Neena et al., 2022 [12]	Prospective case–control study; 36 atropine-treated children and 19 controls	Prospective clinical follow-up with a control group; ocular alignment and near point of convergence were assessed	Non-randomized design; relatively small sample; ocular alignment and binocular function were not the primary focus
Breliant et al., 2023 [13]	Randomized double-masked study; *n* = 46	Randomized and masked design; direct short-term assessment of binocular vision and accommodation after different atropine concentrations	Single-instillation design with short observation window; limited ability to assess longer-term or rare strabismus-related events
Pan et al., 2025 [14]	Secondary analysis of a prospective study; *n* = 205	Prospective 12-month follow-up; repeated accommodation and binocular vision assessments; comparison of 0.05% dosing regimens	Secondary analysis; children with abnormal binocular function or strabismus were excluded; limited applicability to high-risk binocular populations
Santos-Neto et al., 2025 [15]	Randomized interventional study; *n* = 34	Direct assessment of accommodation and vergence parameters after 0.025% and 0.05% atropine	Small sample; short treatment duration; not designed to estimate incidence of manifest strabismus
Iwata et al., 2026 [16]	Case report; *n* = 1	Detailed longitudinal clinical course, including ocular alignment and AC/A ratio before and after discontinuation	Hypothesis-generating only; cannot establish incidence or causality
Kothari et al., 2020 [17]	Case series; *n* = 3	Clinically relevant postoperative intermittent exotropia cases with documented high AC/A ratio and recovery after discontinuation	Small uncontrolled case series; high susceptibility to publication and selection bias; patients had pre-existing binocular vulnerability
Jahan et al., 2021 [18]	Case report; *n* = 1	Detailed clinical course before, during, and after 0.01% atropine exposure	Hypothesis-generating only; prior atropine exposure and baseline binocular status limit causal interpretation
Belbase et al., 2025 [19]	Case report; *n* = 1	Detailed description of ocular alignment, diplopia, stereopsis, and improvement after treatment change	Hypothesis-generating only; cannot separate atropine effect from baseline binocular vulnerability or other clinical factors
Aman and Guyton 2025 [20]	Case report; *n* = 1	Clinically relevant report of acquired esotropia during long-term atropine treatment	Escalating atropine concentrations rather than fixed low-dose monotherapy; should be interpreted separately from standard 0.01–0.05% fixed regimens

## Data Availability

No new data were created or analyzed in this study. Data sharing is not applicable to this article.

## Supplementary Materials

The following supporting information can be downloaded at: https://www.mdpi.com/article/10.3390/children13060818/s1. Table S1: Complete database-specific search strategies.

## Abbreviations

The following abbreviations are used in this manuscript:

AC/A	accommodative convergence/accommodation
AMIXT	0.01% Atropine Eye Drops in Children with Myopia and Intermittent Exotropia trial
ATOM2	Atropine for the Treatment of Myopia 2
D	diopter
DIMS	defocus incorporated multiple segments
IXT	intermittent exotropia
LAMP	Low-Concentration Atropine for Myopia Progression
NPC	near point of convergence
NPA	near point of accommodation
NRA	negative relative accommodation
OK	orthokeratology
PAL	progressive addition lens
PD	prism diopter
PFA	positive fusional amplitude
PFV	positive fusional vergence
PRA	positive relative accommodation
RCT	randomized controlled trial
