# Supplementary material for: Ocular Alignment and Strabismus-Related Findings Associated with Low-Dose Atropine for Myopia Control in Children: A Structured Narrative Review
Source: Children (Basel). 2026 Jun 14;13(6):818. doi: 10.3390/children13060818 (PMC13298291; doi:10.3390/children13060818)
Supplement: Supplementary file 1 [file children-13-00818-s001.zip › children-4327323-supplementary.pdf]

children

MDPI

Supplementary Table S1. Complete database-specific search strategies

Searches were conducted from database inception to 16 April 2026. Searches were restricted to English-language records. No document-type filter was applied.

Database	Item	Details
PubMed/ MEDLINE	Search date	16 April 2026
	Search field	Title/Abstract
	Limit	English
	Records retrieved	109
	Exact search string	(atropine[Title/Abstract]) AND (myopia[Title/Abstract] OR "myopia control"[Title/Abstract] OR "myopia progression"[Title/Abstract] OR "myopia management"[Title/Abstract]) AND ("ocular alignment"[Title/Abstract] OR strabismus[Title/Abstract] OR esotropia[Title/Abstract] OR exotropia[Title/Abstract] OR "accommodative esotropia"[Title/Abstract] OR "convergence excess"[Title/Abstract] OR heterophoria[Title/Abstract] OR phoria[Title/Abstract] OR tropia[Title/Abstract] OR "binocular vision"[Title/Abstract] OR vergence[Title/Abstract] OR convergence[Title/Abstract] OR accommodation[Title/Abstract] OR diplopia[Title/Abstract] OR asthenopia[Title/Abstract] OR "near blur"[Title/Abstract])
Web of Science Core Collection	Search date	16 April 2026
	Search field	Topic search (TS); Title, Abstract, Author Keywords, and Keywords Plus
	Limit	English
	Records retrieved	138
	Exact search string	#1 TS=(atropine)<br>#2 TS=(myopia OR "myopia control" OR "myopia progression" OR "myopia management")<br>#3 TS=("ocular alignment" OR strabismus OR esotropia OR exotropia OR "accommodative esotropia" OR "convergence excess" OR heterophoria OR phoria OR tropia OR "binocular vision" OR vergence OR convergence OR accommodation OR diplopia OR asthenopia OR "near blur")<br>#4 #1 AND #2 AND #3

Abbreviations: TS, Topic search.
